# Newly identified single-nucleotide polymorphism associated with the transition from nonalcoholic fatty liver disease to liver fibrosis: results from a nested case-control study in the UK biobank

**DOI:** 10.1080/07853890.2025.2458201

**Published:** 2025-02-03

**Authors:** Yitong Ling, Yu Xuan Yang, Yan Chun Chen, Jing Hao Wang, Dong Ge Feng, Shi Jian Xiang, Xiaoyu Zhang, Jun Lyu, Sha Sha Li

**Affiliations:** a Department of Neurology, Jinan University First Affiliated Hospital, Guangzhou, China; b Department of Pharmacy, Jinan University First Affiliated Hospital, Guangzhou, China; c School of Pharmacy, Jinan University, Guangzhou, China; d The Guangzhou Key Laboratory of Basic and Translational Research on Chronic Diseases, the First Affiliated Hospital, Jinan University, Guangzhou China;; e Department of Pharmacy, The Seventh Affiliated Hospital, Sun Yat-sen University, Shenzhen, China; f Department of Rheumatology, Affiliated Hospital of Qingdao University, Qingdao, China; g Department of Infectious Diseases, Third Affiliated Hospital, Sun Yat-sen University, Guangzhou, China; h Department of Clinical Research, Jinan University First Affiliated Hospital, Guangzhou, China; i Guangdong Provincial Key Laboratory of Traditional Chinese Medicine Informatization, Guangzhou, China

**Keywords:** Single-nucleotide polymorphism, nonalcoholic fatty liver disease, liver cirrhosis, SAMM50-rs2073080

## Abstract

**Background:**

Genetic factors may have a significant influence on the likelihood of liver fibrosis in individuals with nonalcoholic fatty liver disease (NAFLD). The present study was conducted to explore how single-nucleotide polymorphism (SNP) impacts the development of fibrosis in those suffering from NAFLD.

**Materials and methods:**

Utilizing the UK Biobank dataset, we conducted a nested case-control analysis among NAFLD participants, defining the case group as those with liver fibrosis and cirrhosis during follow-up. For our *in vitro* investigations, we employed the LX-2 human hepatic stellate cell line. Our procedures included cultivating these cells, employing SAMM50-rs2073080 plasmid techniques to enhance the expression of recently discovered SNPs, and conducting biochemical assays. To quantify gene expression, we used real-time PCR with fluorescence detection.

**Results:**

The study analyzed data from 5467 participants (1094 cases and 4373 controls). Genome-wide association analysis identified nine significant loci, including the novel rs2073080 variant, strongly associated with NAFLD-associated hepatic fibrosis. *In vitro* TGF-β modeling revealed significant upregulation of α-SMA and COL1A1, confirming model effectiveness. Oxidative stress markers like elevated malondialdehyde (MDA) and reduced catalase (CAT) and superoxide dismutase (SOD) levels indicated liver damage in the TGF-β group. SAMM50-rs2073080 was upregulated in the NAFLD-associated fibrosis model. *In vitro* experiments on LX-2 cells showed that SAMM50-rs2073080 overexpression led to increased fibrosis, as indicated by higher cellular MDA levels and lower CAT and SOD levels, compared to the vector group.

**Conclusion:**

Our research highlights a significant association of SAMM50-rs2073080 with the progression of NAFLD to hepatic fibrosis, and the *in vitro* experiments further corroborated these findings.

## 1. Introduction

Nonalcoholic fatty liver disease (NAFLD) is presently one of the most prevalent chronic liver diseases globally, with a prevalence rate of about 25% [1]. Most NAFLD has a benign clinical course; however, once simple steatosis progresses to nonalcoholic steatohepatitis (NASH), 25% of patients may progress to liver fibrosis and cirrhosis [2]. Individuals with liver fibrosis grades F2-4 possess a higher risk of liver-related events and mortality, thereby classifying this population as ‘at-risk’ for NASH development [3]. Characterized by the accumulation of extracellular matrix in the liver, liver fibrosis arises due to various types of chronic liver damage and is closely associated with cirrhosis and hepatocellular carcinoma [4]. Approximately, 90% of hepatocellular carcinoma cases stem from cirrhosis, with an estimated annual death toll of 1 million individuals worldwide, a number that continues to rise [5]. Liver fibrosis progression is a crucial NAFLD outcome determinant, though no effective treatment for liver fibrosis currently exists, it can be reversed in its early stages. Consequently, liver fibrosis represents a promising therapeutic target for preventing liver cancer development. The purpose of this study was to explore the mechanism of the progression of NAFLD to liver fibrosis, and it was expected that new targets can effectively inhibit the progression of NAFLD to liver fibrosis through this study and provide a new direction for the treatment of liver fibrosis.

The development of NAFLD is shaped by a combination of environmental influences and genetic predispositions, with its heritability ranging from 22% to 50% [6,7]. The application of genome-wide association study (GWAS) technology has identified multiple genetic polymorphisms, such as PNPLA3 I148M, TM6SF2 E167K, and MBOAT7 rs641738, as major predictors of NAFLD development and progression [8]. These variants affect lipid accumulation and metabolism in the liver, as well as mitochondrial function and metabolic reprogramming, increasing the risk of developing a more severe form of liver disease. In addition, previous GWAS have found that the SAMM50 variant and several single-nucleotide polymorphisms (SNPs) such as rs738491, rs3761472, and rs2073082 are linked to increased NAFLD risk [9–11]. Clinical research supports these findings, with the rs738491 variant also tied to fibrosis in a Japanese cohort study[9]. Such discoveries have emphasized the pivotal role of SNPs in NAFLD’s pathogenesis. Nonetheless, the generalizability of these findings is constrained by the relatively small cohorts examined, necessitating research with larger sample sizes.

Leveraging the extensive UK Biobank cohort, this study employs a nested case-control design to explore the genetic and environmental factors contributing to the transition from NAFLD to liver fibrosis. By defining phenotypes, conducting GWAS, and employing advanced cell culture and biochemical assays, this study aims to unravel the genetic mechanisms and identify potential therapeutic targets for the transition from NAFLD to liver fibrosis.

## 2. Materials and methods

### 2.1. Participants and phenotype definition

This research utilized the UK Biobank, a vast, prospective cohort study, recruiting individuals aged 40-69 from England, Wales, and Scotland between 2006 and 2010. It gathered data on sociodemographic aspects, lifestyle, health, and genetics. Approval for the UK Biobank was granted by the North West Multi-Centre Research Ethics Committee. Our research utilized the UK Biobank resource under application number 62017. Because all data come from public databases, the requirement for approval was waived by the Human Ethics Committee of Jinan University First Affiliated Hospital.

The study was designed as a nested case-control study. Within the UK Biobank cohort, the fatty liver index (FLI) was computed for each participant to delineate hepatic steatosis when the FLI exceeded 60. The FLI, an established and broadly acknowledged indicator of hepatic lipid accumulation in humans, is calculated through an equation encompassing factors like triglycerides, body mass index (BMI), gamma-glutamyl transferase, and waist measurement [12]. Participants with baseline alcohol-related liver disease, fibrosis, cirrhosis of the liver, liver failure, liver cancer, or lacking genetic data were excluded, resulting in 171,196 remaining NAFLD participants. Cases were defined as participants with fibrosis and cirrhosis of the liver during the follow-up, which was determined *via* the UK Biobank’s health-related outcomes, including ­primary care data, hospital inpatient data, and self-reported medical conditions. Controls were matched 4:1 for age, sex, BMI, education, ethnicity, smoking, alcohol consumption, sleep duration, concurrent hypertension, diabetes, cardiovascular disease, and hypercholesterolemia at recruitment from the NAFLD-diagnosed participants.

### 2.2. Genome-wide association analysis

In the genotyping process of the UK Biobank, two distinct Affymetrix arrays were employed. The UK BiLEVE Axiom array facilitated the analysis of approximately 50,000 participants, whereas the remaining 450,000 participants were analyzed with the UK Biobank Axiom array. Despite using different platforms, both arrays effectively covered a similar range of over 800,000 SNPs, with a shared genotype coverage of around 95%. The UK Biobank had centralized quality control of genotype data [13]. Further stringent quality control was applied using PLINK v1.9. Markers with a missingness rate of 2% or higher were omitted. Any markers not adhering to Hardy-Weinberg equilibrium, defined by a threshold of *p* < 1 × 10^−6^, were excluded. Additionally, markers with a minor allele frequency below 1% were also not included. Association with liver fibrosis was analyzed through logistic regression, adjusting for age, sex, BMI, education, ethnicity, smoking, alcohol consumption, sleep duration, concurrent hypertension, diabetes, cardiovascular disease, hypercholesterolemia, and the first 20 genetic principal components. For the genome-wide analysis significance was set at *p* < 5 × 10^−8^.

### 2.3. Cell culture

The hepatic stellate cell line (LX-2) was obtained from Procell Life Science & Technology Co. Ltd. (Wuhan, China) and was cultured in Dulbecco’s modified Eagle’s medium (DMEM) from GIBCO located in Grand Island, NY. The DMEM was supplemented with 10% FBS obtained from Gibco in the United States. Additionally, it contained 10 × 105 U·L^−1^ penicillin and 100 mg·L^−1^ streptomycin. The cells were maintained at a temperature of 37 °C with a CO_2_ concentration of 5%. Our cell experiment was to culture three batches of cells, which were derived from three batches of different cells, and each cell sample was tested in three replicates. Due to their highly dynamic and complex metabolic activities, cells in the logarithmic growth phase provide abundant materials for the study of metabolic pathways, signaling, etc. within cells. For the experiments, cells in the logarithmic growth phase were selected and cell counting was used to determine the logarithmic growth phase of cells [14,15]. To analyze the expression of profibrogenic genes, the cells were incubated with a concentration of 10 ng/mL TGF-β for 48 h to induce cellular liver fibrosis, which was consistent with the description in other study [16]. Liver function indicators alanine aminotransferase (ALT) and aspartate aminotransferase (AST), as well as liver fibrosis indicators α-SMA and collagen I, were used to determine the formation of liver fibrosis.

### 2.4. Overexpression of newly identified SNP by plasmid treatment

Firstly, the SAMM50-rs2073080 overexpression plasmid was designed. When the cell density was determined to be about 90%, the fresh medium was replaced. Using a plasmid transfection kit for transfection, the transfection complex was added to the medium. The plate was gently shaken while adding and then transferred to the incubator for 48 h. Subsequently, the cells were collected and preserved at a temperature of −80 °C until further use.

### 2.5. Biochemistry assays

The concentration values of superoxide dismutase (SOD), catalase (CAT), malondialdehyde (MDA) and alanine aminotransferase (ALT, C009-2-1), aspartate aminotransferase (AST, C010-2-1) of the human hepatic stellate cells (LX-2) and the SAMM50-rs2073080 overexpression cells were determined according to the instructions of the diagnostic kits, which were acquired from the Nanjing Jiancheng Bioengineering Institute (Nanjing, China).

### 2.6. Real-time PCR analysis

RT-qPCR was employed to analyze the gene expression in human hepatic stellate cells (LX-2) and AMM50-rs2073080 overexpression cells. Total RNA from both the human hepatic stellate cells (LX-2) was isolated using a Trizol reagent. The RNA content was determined by measuring the optical density at 260 nm. To synthesize cDNA, HiScript II Q RT SuperMix for qPCR (cat no. R223-01, Vazyme, Nanjing, China) was used. The resulting cDNA was then utilized as a template and quantified using the BioEasy Master Mix Kit (cat no. BSB25L1B, SYBR Green, High ROX) and a real-time PCR detection system (Line Gene 9600 Plus, Bioer Technology, China). GAPDH was used as the control to evaluate the mRNAs content of α-SMA and collagen I. The expressions of mRNAs were analyzed using the 2^−ΔΔCt^ method. The information on primers is shown in Table 1.

**Table 1.** Information of primers.

Gene (Human)	Forward (5′–3′ sequence)
GAPDH-F	ACCCTTAAGAGGGATGCTGC
GAPDH-R	CCCAATACGGCCAAATCCGT
α-SMA -F	GCCATCTTTCATTGGGATGGA
α-SMA-R	CCCCTGACAGGACGTTGTTA
COL1A1-F	CGATGGATTCCCGTTCGAGT
COL1A1-R	GAGGCCTCGGTGGACATTAG
SAMM50-rs2073080-HUMAN-F	AGTGGAGGTCCTGGGTGAAA
SAMM50-rs2073080-HUMAN-R	AAGGGAGGATTTCATGGGGG

### 2.7. Statistical analysis

In our study, we cultured three separate batches of cells, each derived from a distinct source. Furthermore, we conducted three replicates for each cell sample during the testing phase, in line with a previous study [17]. Statistical analyses were performed to examine the data, and the mean ± standard deviations (SD) were expressed. The software used for these analyses included GraphPad Prism 9.0 (GraphPad Software, Inc; San Diego, CA, USA) and R (version 4.2.3). To compare multiple groups, one-way ANOVA and Tukey’s post hoc test were performed as statistical methods in this study. A significance level of *p* < 0.05 was deemed statistically significant for the tests conducted, except for the genome-wide analysis.

## 3. Result

### 3.1. Demographic characteristics

During the median follow-up period of 12.7 years, a total of 1,094 cases were identified, with 4,373 controls matched at a 4:1 ratio. The median age was 61 years and 64.9% were male. Table 2 showed that most participants were overweight, as indicated by a median BMI of 31.51. A minority of the population had a college or university degree (18.7%), while a significant majority were of White ethnicity (96.3%). Regarding lifestyle factors, most currently consumed alcohol (87.8%). Over half had hypertension (55.5%), a notable percentage had high cholesterol (23.6%) and diabetes (29.0%). The baseline characteristics between cases and controls did not show significant differences (all *P* value > 0.05).

### 3.2. Genome-wide association analysis

We identified nine genome-wide significant loci (*p* < 5 × 10^−8^) associated with liver fibrosis (Table 3). Notably, the rs2073080 variant was first identified in patients with NAFLD-associated hepatic fibrosis (*p* = 7.32 × 10^−15^).

**Table 2.** The number of controls and patients with liver fibrosis in a nested case-control study from the participants in the UK biobank.

Characteristics	Overall	Case group	Control group	*P* value
***N***	5467	1094	4373	
**Sex (*n*, %)**				0.656
Female	1920 (35.1)	391 (35.7)	1529 (35.0)	
Male	3547 (64.9)	703 (64.3)	2844 (65.0)	
**Age (Median, IQR)**	61.00 [54.00, 65.00]	61.00 [54.00, 65.00]	61.00 [54.00, 65.00]	0.794
**BMI (Median, IQR)**	31.51 [29.00, 34.95]	31.61 [28.50, 35.72]	31.50 [29.09, 34.78]	0.920
**Sleep duration (Median, IQR)**	7.00 [6.00, 8.00]	7.00 [6.00, 8.00]	7.00 [6.00, 8.00]	0.642
**Education (*n*, %)**				0.985
College/University degree	1023 (18.7)	204 (18.6)	819 (18.7)	
Others	4444 (81.3)	890 (81.4)	3554 (81.3)	
**Ethnic (*n*, %)**				0.983
White	5264 (96.3)	1054 (96.3)	4210 (96.3)	
Others	203 (3.7)	40 (3.7)	163 (3.7)	
**Smoking (*n*, %)**				0.869
Never or previous	4574 (83.7)	913 (83.5)	3661 (83.7)	
Current	893 (16.3)	181 (16.5)	712 (16.3)	
**Alcohol (*n*, %)**				0.708
Never or previous	669 (12.2)	138 (12.6)	531 (12.1)	
Current	4798 (87.8)	956 (87.4)	3842 (87.9)	
**Hypertension (*n*, %)**	3033 (55.5)	601 (54.9)	2432 (55.6)	0.712
**High cholesterol (*n*, %)**	1289 (23.6)	251 (22.9)	1038 (23.7)	0.712
**Cardiovascular disease (*n*, %)**	810 (14.8)	162 (14.8)	648 (14.8)	1.000
**Diabetes (*n*, %)**	1584 (29.0)	315 (28.8)	1269 (29.0)	0.913

BMI: body Mass index.

**Table 3.** Genome-wide significant loci associated with progression from NAFLD to liver fibrosis.

SNP	CHR	BP	A1	BETA	OR	P value
rs738409	22	44324727	G	0.5411	1.718	2.78E−24
rs12483959	22	44325996	A	0.5406	1.717	1.18E−19
rs2281135	22	44332570	A	0.5268	1.693	4.57E−19
Affx-19716376	22	44332888	TC	0.5317	1.702	2.8E0−19
rs3761472	22	44368122	G	0.504	1.655	6.66E−17
rs3827385	22	44388817	C	0.4609	1.585	2.93E−15
rs2143571	22	44391686	A	0.4753	1.608	5.37E−16
rs2073080	22	44394402	T	0.4564	1.578	7.32E−15
rs1007863	22	44395451	C	0.2885	1.334	4.53E−09

Chr: chromosome; BP: base position; A1: effect allele; OR: odds ratio.

### 3.3. Liver fibrosis was molded with TGF- β

**Figure 1.**
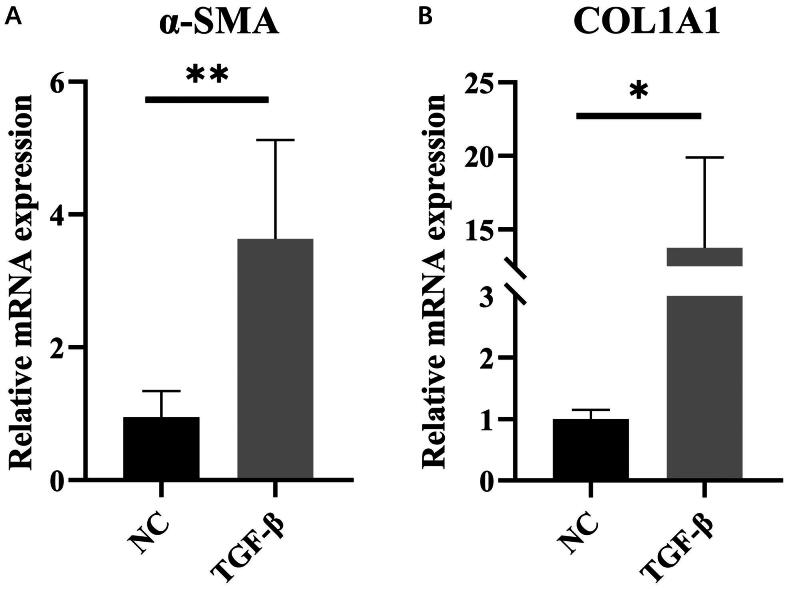
mRNA Expressions of α-SMA and COL1A1. (A) The relative mRNA expression levels of α-SMA were determined by qRT-PCR. (B) The relative mRNA expression levels of COL1A1 were determined by qRT-PCR. The data are expressed in the format of mean ± SD (*n* = 3). *p < 0.05, **p < 0.01, and ***p < 0.001 vs. TGF-β.

To determine the success of the modeling, we conducted a validation process. Following q-PCR analysis, it was observed that the expression levels of α-SMA and COL1A1 were significantly augmented in the model group when compared to the control group, thereby confirming the efficacy of the cellular modeling procedure (Figure 1). In order to assess the degree of liver damage inflicted upon LX-2 cells, the levels of oxidative stress were measured. Our findings revealed elevated MDA levels in the TGF-β treatment group as compared to the NC group, whereas CAT and SOD levels were found to be lower in the TGF-β treatment group when compared to the NC group.

### 3.4. SAMM50-rs2073080 was upregulated in the cell NAFLD-associated fibrosis model

**Figure 2.**
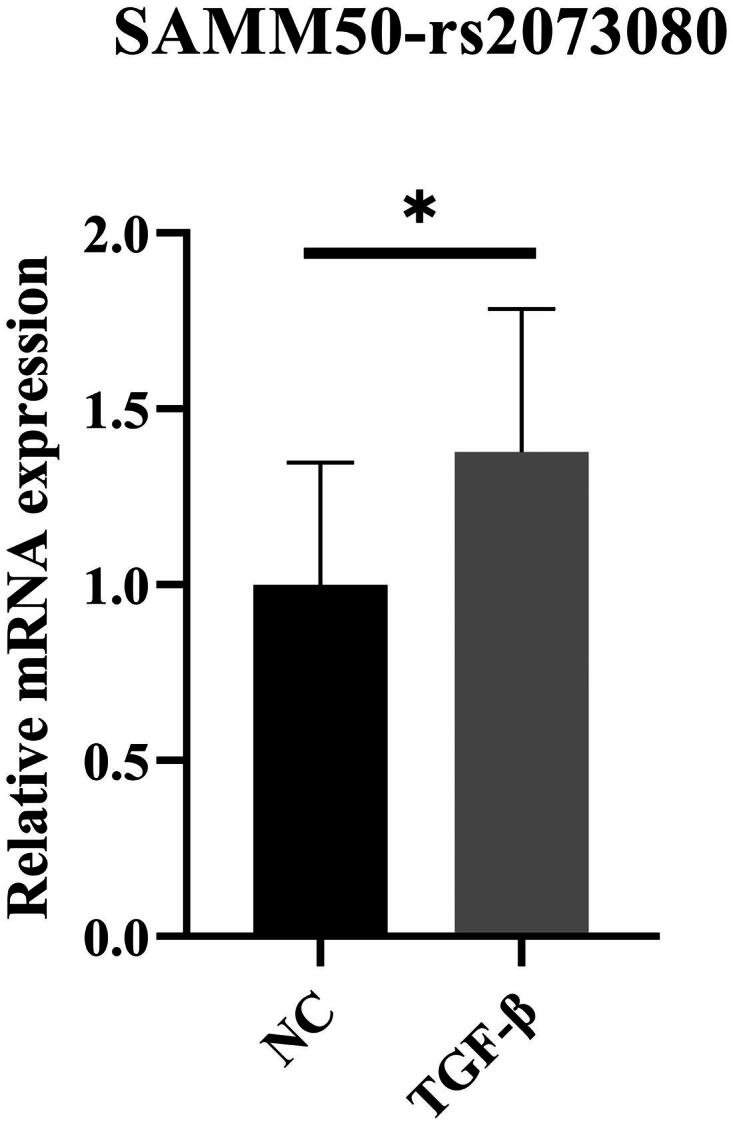
SAMM50-rs2073080 expression in NC and TGF-β. The relative mRNA expression levels of SAMM50-rs2073080 were determined by qRT-PCR. The data are expressed in the format of mean ± SD (*n* = 3). *p < 0.05, **p < 0.01, and ***p < 0.001 vs. TGF-β.

SAMM50-rs2073080 functions as the focal SNP. In addition, we conducted q-PCR analysis on hepatic stellate cells derived from humans in an *in vitro* cellular examination. When compared to the control group, the q-PCR results demonstrated that the expression level of the SAMM50-rs2073080 gene was elevated in the model group cells (Figure 2).

### 3.5. SAMM50-rs2073080 impacts liver fibrosis

**Figure 3.**
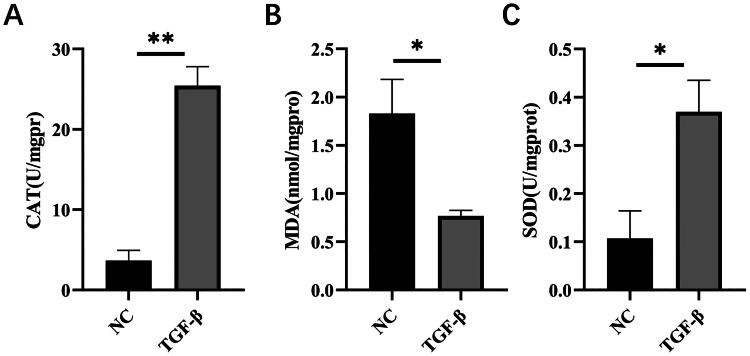
SAMM50-rs2073080 activation of LX-2 cells. (A) CAT content in LX-2 cell, (B) MDA content in LX-2 cell, (C) SOD content in LX-2 cell. The data are expressed in the format of mean ± SD (*n* = 3). *p < 0.05, **p < 0.01, and ***p < 0.001 vs. TGF-β.

In order to further evaluate the impact of the SAMM50-rs2073080 variant on liver fibrosis, a series of *in vitro* experiments were conducted using LX-2 cells. To achieve overexpression of SAMM50-rs2073080 in LX-2 cells, a plasmid was engineered for this purpose, and the success of overexpression was confirmed. In order to gauge the extent of fibrosis in LX-2 cells, the levels of oxidative stress were measured. Correspondingly, the overexpression group exhibited elevated cellular MDA levels compared to the vector group, whereas the overexpression group demonstrated diminished levels of cellular CAT and SOD in comparison to the vector group (Figure 3).

### 3.6. SAMM50-rs2073080 overexpression plasmid was successfully constructed

**Figure 4.**
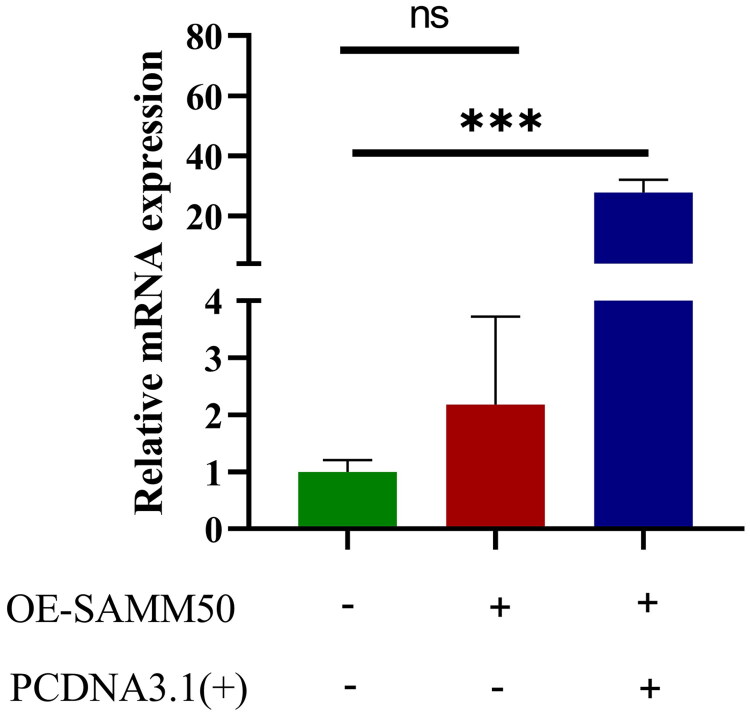
SAMM50-rs2073080 overexpression plasmid was successfully constructed. The data are expressed in the format of mean ± SD (*n* = 3). *p < 0.05, **p < 0.01, and ***p < 0.001.

SAMM50-rs2073080 was overexpressed in LX-2 cells, and the overexpression efficiency of SAMM50-rs2073080 was detected by qPCR, and the results showed that the expression level of SAMM50-rs2073080 in the SAMM50-rs2073080 OE group was significantly higher than that in the normal group, indicating that the cells were successfully transfected, as shown in Figure 4.

### 3.7. Overexpression of SAMM50-rs2073080 affected liver function indexes

**Figure 5.**
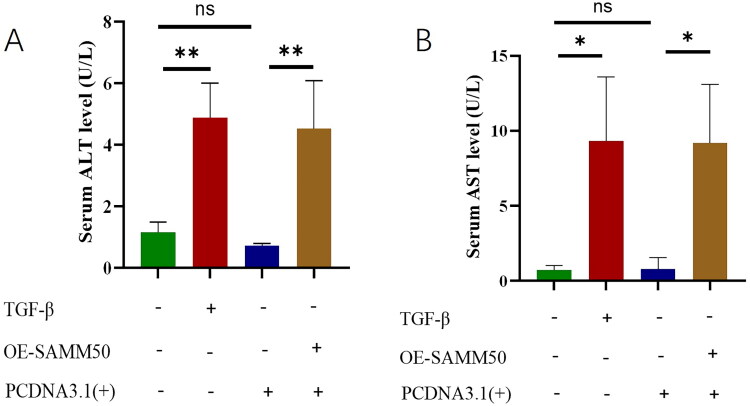
Overexpression of SAMM50-rs2073080 affected liver function indexes. (A) ALT content in LX-2 cell, (B) AST content in LX-2 cell. The data are expressed in the format of mean ± SD (*n* = 3). *p < 0.05, **p < 0.01, and ***p < 0.001.

**Figure 6.**
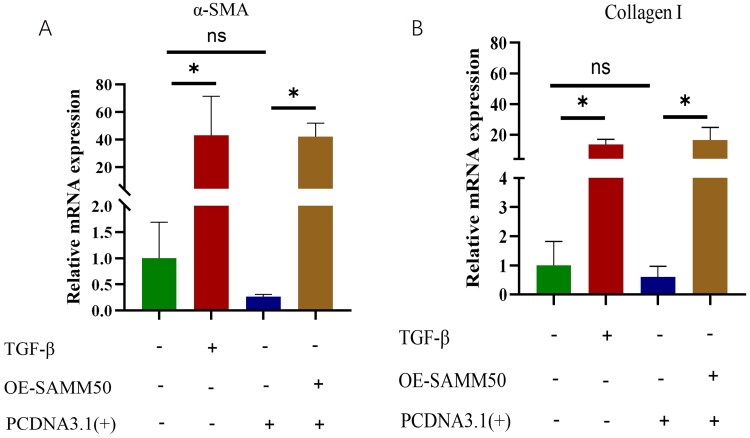
Overexpression of SAMM50-rs2073080 affected indicators of liver fibrosis. The relative mRNA expression levels of α-SMA (A) and collagen I (B) were determined by qRT-PCR. The data are expressed in the format of mean ± SD (*n* = 3). *p < 0.05, **p < 0.01, and ***p < 0.001.

ALT and AST were significantly upregulated in Lx2 cells overexpressed with SAMM50-rs2073080, suggesting that SAMM50-rs2073080 overexpression could lead to liver dysfunction (Figure 5). In addition, liver fibrosis-related mRNAs such as α-SMA and collagen I were significantly up-regulated in LX-2 cells overexpressed by SAMM50-rs2073080, indicating that SAMM50-rs2073080 overexpression would accelerate the progression of liver fibrosis (Figure 6).

### 3.8. Overexpression of SAMM50-rs2073080 affected oxidative stress indexes

**Figure 7.**
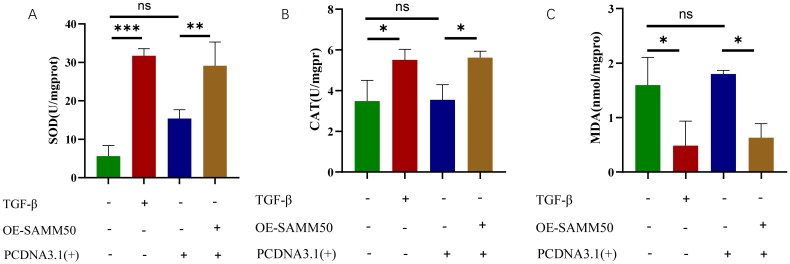
Overexpression of SAMM50-rs2073080 affected indicators of oxidative stress. (A) SOD content in LX-2 cell, (B) CAT content in LX-2 cell, (C) MDA content in LX-2 cell. The data are expressed in the format of mean ± SD (*n* = 3). *p < 0.05, **p < 0.01, and ***p < 0.001.

The oxidative stress indexes SOD and CAT were ­significantly up-regulated and MDA significantly down-regulated in LX-2 cells overexpressed with SAMM50-rs2073080 (Figure 7), indicating that in hepatic stellate cells cultured with SAMM50-rs2073080 after activation with TGF-beta, there is reduced lipid peroxidation and increased activity of CAT and SOD.

## 4. Discussion

Our study yielded pivotal insights into the genetic mechanisms underlying liver fibrosis. The genome-wide association analysis identified nine loci significantly associated with liver fibrosis, highlighting the rs2073080 variant as particularly significant in NAFLD-associated hepatic fibrosis. *In vitro* studies using human hepatic stellate cells indicated the upregulation of SAMM50-rs2073080 in the liver fibrosis model. Additionally, our findings demonstrated that treatment with SAMM50-rs2073080 plasmid in the model reduced ALT, AST, and oxidative stress markers, suggesting its potential therapeutic role in liver fibrosis management.

This study leveraged the UK Biobank database and employed a nested case-control design. The vast and diverse participant cohort within the UK Biobank provided a rich source of data, enhancing the generalizability of our findings to broader populations. The nested case-control design, with its meticulous matching of controls to cases, strengthens the internal validity of our study by minimizing potential confounders. This approach allows for a more nuanced exploration of genetic associations with liver fibrosis within the context of NAFLD. The combination of a large-scale database and a well-structured study design positions our investigation at the forefront of unraveling the intricate genetic underpinnings of liver fibrosis, fostering robust and reliable conclusions.

The SAMM50 gene and its encoded protein, SAM50, play crucial roles in the mechanical complex responsible for sorting and assembling components on the outer membrane of mitochondria. This complex is vital for preserving the stability of mitochondrial DNA, the respiratory chain complex, and the structural integrity of the mitochondrial crest [9,10,18]. Furthermore, it is involved in regulating mitophagy, a process essential for removing damaged mitochondria [19]. Notably, deficiency of SAM50 has been linked to membrane restructuring and impaired mitochondrial function. Additionally, the reduced ability to remove reactive oxygen species (ROS), accompanied by lipotoxicity and hepatocyte damage, contributes to the progression of NAFLD [11].

There is extensive scientific evidence supporting the notion that the SAMM50-rs2073080 risk allele poses a significant and autonomous risk for NAFLD. Its impact remains unaffected by variables such as gender, age, BMI-Z scores, other metabolic factors, and it remarkably influences both the onset and severity of NAFLD in the elderly and younger population [20,21]. Investigative studies have demonstrated that impaired mitochondrial function can trigger the progression of liver fibrosis by prompting the expression of TGF-β through molecular markers linked to mitochondrial impairment [22]. Nevertheless, it is important to note that the current body of research concerning the relationship between SAMM50-rs2073080 and liver fibrosis is limited.

Oxidative stress, characterized by imbalanced ROS or reactive nitrogen species (RNS) resulting from aerobic metabolism and antioxidant defense, refers to a pathological condition. The body’s SOD serves as a scavenger for superoxide radicals. If there is an excessive production of ROS or insufficient clearance, the phospholipid membrane binds to create lipid peroxides. The primary byproduct of its breakdown is MDA [23]. In the initiation and progression of liver fibrosis, oxidative stress plays a crucial role [24]. ROS and MDA have the ability to assault membrane proteins, mitochondrial DNA, thereby causing hepatocyte necrosis, apoptosis, and promoting liver fibrosis [25]. Both SOD and MDA serve as significant indicators for assessing the levels of oxidative stress. Upon formation, there was a decrease in SOD and CAT content, accompanied by an increase in MDA content.

Hematopoietic stem cells (HSCs) are an important component of NAFLD-associated liver fibrosis cell models, and they play a key role in the development of inflammation and fibrosis in the disease. HSCs are central to the formation of liver fibrosis, and they are activated in the early stages of the disease and worsen as the disease progresses from NAFLD to NASH [26]. The LX-1 and LX-2 are different cell lines derived from LX cells. Detailed characterization has confirmed that these cell lines maintain important characteristics related to cytokine signaling, gene expression in ­neurons, retinoid metabolism, and fibrogenesis. Consequently, these cell lines are highly suitable for conducting culture-based studies on liver fibrosis. In comparison to LX-1 cells, LX-2 cells can survive in a serum-free medium supplemented with 0.2% BSA. Both LX-1 and LX-2 cells exhibit a response to TGF-β1, a major cytokine associated with fibrosis in the liver. The presence of elevated TGF-β1, levels has been observed in animal models of liver fibrosis as well as in patients suffering from chronic liver disease. Notably, LX-2 cells possess a distinct advantage in terms of their ability to be efficiently transfected, with a transfection efficiency of 30% using commercial reagents (such as Fugene). In contrast, LX-1 cells and other astrocytic culture systems reported thus far have shown a transfection efficiency of less than 1 [27]. Given these advantages, we have selected LX-2 cells for use in our experiments due to their high transfection efficiency and resistance to serum-free conditions.

TGF-β, a cluster of TGF-β groups, governs cell growth and differentiation. It is naturally found in healthy liver tissue but exhibits heightened expression throughout progressive liver disease stages, including fibrosis [28]. Typically, a segment of TGF-β remains stored in the extracellular matrix (ECM) as TGF-β-binding protein (LTBP) or a complex. Upon activation, TGF-β regulates ECM remodeling, facilitates fibroblast to myofibroblast transition, and influences other cell types involved in fibrosis, such as epithelial, endothelial, or macrophage cells. These actions play a crucial role in the fibrotic process [29]. To induce cell activation, TGF-β was chosen as the stimulus. Following TGF-β modeling, RNA analysis using RT-qPCR determined the levels of mRNA expression for liver fibrosis markers, namely α-SMA and Collagen I. The findings demonstrated an increase in the expression of these markers in the modeled group, suggesting the successful implementation of the modeling process.

Several limitations should be considered in the interpretation of our study findings. Firstly, the utilization of a blood marker equation for defining liver steatosis may introduce a degree of imprecision. While liver biopsy stands as the gold standard for diagnosing hepatic steatosis, its impracticality in the context of a large-scale epidemiological study necessitated the use of blood markers. Despite its widespread application in similar studies, the inherent limitations of this approach should be acknowledged [30]. Secondly, our study predominantly focused on individuals of European ancestry, necessitating caution in generalizing our results to populations of diverse ethnic backgrounds. Furthermore, due to the lack of expression of this genotype in the animal system, our research lacks validation from animal models. Without validation from animal models, the complexity of the biological mechanisms underlying hepatic steatosis and its progression may not be fully captured. Future research endeavors should incorporate this dimension to enhance the translational relevance of our findings. In addition, there is a lack of research on mitochondrial function *in vitro* to link oxidative stress, and future studies will further explore mitochondrial function. Lastly, the study does not delve into the detailed exploration of downstream mechanisms governing the observed genetic associations. While we have identified potential risk loci, understanding the intricate molecular pathways and interactions contributing to liver steatosis requires further investigation. The absence of a comprehensive exploration of these mechanisms limits the depth of our current understanding and highlights the need for subsequent studies to unravel the molecular intricacies associated with hepatic steatosis.

## 5. Conclusion

Our research highlights the significant association of SAMM50-rs2073080 with the progression of NAFLD to hepatic fibrosis. This sequence is located in a non-coding region, but it has been reported to influence disease progression [2, 21]. The GWAS revealed crucial loci that predispose individuals to liver fibrosis. The *in vitro* experiments further corroborated these findings, demonstrating the upregulation of SAMM50-rs2073080 in a TGF-β induced fibrosis model. Importantly, the intervention with SAMM50-rs2073080 plasmid showcased a potential therapeutic pathway, as evidenced by reduced liver injury markers and oxidative stress. These insights into the genetic mechanisms of NAFLD and its progression to fibrosis could significantly influence future clinical approaches and therapeutic strategies, offering a more personalized and effective treatment for patients with this condition.

## Data Availability

The UK Biobank data related to this study are available at UK Biobank resource (https://www.ukbiobank.ac.uk/). The experimental data that support the findings of this study are available from the corresponding author, SL, upon reasonable request.
